# Building capacity for translational team science: digital badging as a pathway to professionalization

**DOI:** 10.3389/fpsyg.2026.1771863

**Published:** 2026-04-10

**Authors:** Kristine M. Glauber, Whitney Sweeney, Heather A. Billings, Mayla R. Boguslav, Andrew T. Fox, Madison Hartstein, Laura Hildreth, Diana Lee-Chavarria, Rebecca Lobb, Wayne T. McCormack, Angela Mendell, Jennifer R. Molano, Heather J. Risser, Allan R. Brasier

**Affiliations:** 1 Clinical and Translational Science Institute, Duke University School of Medicine, Durham, NC, United States; 2 Institute for Clinical and Translational Research, School of Medicine and Public Health, University of Wisconsin-Madison, Madison, WI, United States; 3 Center for Clinical and Translational Science, Mayo Clinic, Rochester, MN, United States; 4 Southern California Clinical and Translational Science Institute, Los Angeles, CA, United States; 5 Frontiers Clinical and Translational Science Institute at the University of Kansas Medical Center, Kansas City, KS, United States; 6 Northwestern University Clinical and Translational Sciences Institute, Northwestern University, Chicago, IL, United States; 7 University of Cincinnati College of Medicine, Center for Clinical & Translational Science & Training, Cincinnati, OH, United States; 8 South Carolina Clinical & Translational Research Institute, Medical University of South Carolina, Charleston, SC, United States; 9 Clinical and Translational Science Institute, Boston University, Boston, MA, United States; 10 Clinical & Translational Science Institute, University of Florida College of Medicine, Gainesville, FL, United States; 11 Department of Psychiatry and Behavioral Sciences, Feinberg School of Medicine, Northwestern University, Chicago, IL, United States; 12 Department of Medicine, School of Medicine and Public Health, University of Wisconsin-Madison, Madison, WI, United States

**Keywords:** competency-based training, digital badges, interdisciplinary collaboration, micro credentials, professional development, team science, translational science, workforce development and training

## Abstract

Effective translation relies on high performance teams integrating diverse disciplines to bring new drugs, medical devices, diagnostics or behavioral interventions into improved health. Despite substantial advancements informed by the Science of Team Science (SciTS), challenges remain in effective translational team (TT) operations, while limited institutional, scholarly, and practitioner legitimacy of team science expertise constrains efforts to address them. Here, we describe an essential member of the TT, the Team Science Professional (TSP). Serving as a team educator and facilitator of team interventions, the TSP plays a pivotal role in facilitating effective team performance. Current challenges faced by this nascent profession are defining career pathways and establishing competencies needed for expert performance. To address these challenges, the authors, representing a spectrum of academic universities organized as a special interest group (SIG) of the Association of Clinical and Translational Science (ACTS), propose a suite of individualized, standardized and pathway-selective micro-credentials (digital badges). Digital badges in “Team Science (TS) Fundamentals,” “TS Practitioner,” “TT Trainer” and “TT Intervention Expert” are described, along with preliminary feasibility and usability evaluations of the submission process. These stackable credentials provide a customizable, portable recognition of skills and help advance career pathways for TSPs. In so doing, the Digital Badge Initiative will enhance the capacity of translational science “Hubs” such as those supported by Clinical and Translational Science Awards to conduct effective and rigorous translation. Moving forward, the ACTS TSP SIG will be examining the impact of digital badging on standardization and its effect on career recognition and retention.

## Introduction

Over the past century, the complexity of scientific and societal challenges has grown substantially and steadily. These “wicked problems” require solutions beyond the scope of any single discipline (Rittel and Webber, 1973). Team Science (TS), or collaborative research that integrates diverse expertise, is uniquely positioned to successfully address such problems, leading to impactful solutions that are more likely to be cited, adopted or licensed. Consequently, cross-disciplinary team approaches are now the norm across the biomedical sciences and engineering fields (Wuchty et al., 2007). Within healthcare, specifically translational research, teamwork is vital in developing successful treatments, diagnostics, interventions, and improving the efficiency, effectiveness and equity of the translational process (Vogel et al., 2023). However, research consistently shows that effective team performance is not merely the result of bringing together the “right: experts, but requires a complex interplay of appropriate attitudes, behaviors, and skills among team members (Sweeney et al., 2023; Lotrecchiano et al., 2020; Bisbey et al., 2021) to ensure their satisfaction and effective team functioning (Dai, 2020).

While there is a growing theoretical and evidence base to support Team Science, a persistent challenge lies in the misalignment between how researchers are trained, and subsequently how team science is practiced, supported, and evaluated (Hall et al., 2018). Many researchers are initially trained within a single-discipline approach. This may provide anecdotal experience in team-based research; however, it rarely provides scientifically rigorous, evidence-informed training in effective cross-disciplinary collaboration. With the acceleration of more scientific efforts requiring a team science approach, there is an increasing demand for standards of knowledge and competence in its practice, education, and evaluation (National Academies of Sciences et al., 2025). A new professional identity that has emerged from this demand is the Team Science Professional (TSP). TSPs–those who practice, support, educate, evaluate, and lead team-based research—are emerging as critical drivers of successful clinical and translational science in a variety of cross-disciplinary settings (Vogel et al., 2023; NCATS, 2024). TSP expertise is increasingly vital to helping teams function effectively to navigate the complexities of the translational pipeline. Clearly articulating the core knowledge and competencies that define TSP expertise is essential—not only to ensure that teams effectively leverage these skills, but also to align with evolving funding priorities that emphasize collaboration, integration, and impact. Establishing shared standards for TSP expertise will enable teams to more efficiently translate discoveries into real-world outcomes (Lotrecchiano et al., 2020; Brasier et al., 2023a,b).

Without standardized TSP competencies, training and professional development opportunities remain inconsistent, fragmented, and constrained by local resources. Many TSPs have developed their competence through “trial by fire,” resulting in a patchwork of knowledge, specialization, and limited recognition or utilization of their contributions by research teams that may not fully understand the scope or value of TSP contributions (Lotrecchiano et al., 2020; Mendell et al., 2024). Further, institutions frequently struggle to support the boundary-spanning nature of TSP roles, often constraining TSP scope due to university disciplinary and administrative structures. This lack of a coherent infrastructure for developing and recognizing TSP fluency undermines both professional legitimacy and institutional efforts to build and maintain high-functioning cross-disciplinary teams. In response to these challenges and the limitations of local resources, TSPs mobilized to create and evaluate a coordinated national effort to establish shared standards, expand access to training, and strengthen professional identity within the broader translational research ecosystem.

## Team science professionals form a special interest group

The Association for Clinical and Translational Science (ACTS) is an organization dedicated to bolstering translational Hubs at academic medical centers across the country supported by NIH Clinical and Translational Science Awards (CTSA) and Institutional Development Award Networks for Clinical Translational Research (IdeA-CTR) programs referred to from here on as Hubs. As one part of its mission, ACTS promotes career development of all members of the translational team through Special Interest Groups (SIGs) that include Clinical Research Professionals, Evaluators, Biostatisticians, Informaticians, Research Operations and Administrators, and others. SIG members are self-identified and composed of faculty, staff, and trainees engaged in all aspects of clinical and translational science (CTS) (ACTS, 2025).

The Team Science Professionals (TSP) SIG was created in early 2024 with the goal to “advance the practice of translational Team Science by developing a career pathway for Team Science professionals – both educators and scholars (ACTS, 2025).” Other objectives were to curate standardized training and develop career pathways for those who recognize themselves as TSPs, with the priority to implement a national credential (digital badge) in Team Science.

## Digital badging for team science professionals

It is critical for individuals within a profession to demonstrate established and specific competencies and acquired skills. In the evolving field of Team Science, one promising approach is to share effective education and training strategies that build the necessary knowledge base and enhance team performance across multiple contexts. When paired with a viable infrastructure and a sustainable operational model (including evaluation), this exchange of capacity-building resources can foster expanded professional development opportunities for TSPs and the institutions that employ them.

A digital badge is one such way of both providing and proving relevant, essential expertise in specific skills and competencies. Digital badges are electronic micro-credentials that verify completion of specific trainings and/or the mastery of related skills, offering a modular, scalable way to recognize competencies (Lee-Chavarria et al., 2023). They can be applied to–and earned–through a variety of formats, including online training modules, workshops, and seminars. Digital badges offer a versatile and customizable approach to professional development, enabling individuals to tailor their training to align with their personal career goals and the specific needs of their professional context. They are “stackable,” enabling learners to tailor trainings to their career goals, building on each learning with badges for subsequent training (Norman et al., 2024). Given the rapid evolution of the field of Team Science, digital badges meet the demands for validated learning that aligns with the dynamic roles that TSPs fill.

Digital badges enable users to not only build a professional profile to promote and advance their work as TSPs but also raises the visibility of TSP expertise across the translational research ecosystem. Digital badges are verifiable, non-transferable credentials that can be publicly displayed on professional platforms to document an individual's expertise (e.g., LinkedIn profiles or institutional bios). Embedded metadata specifies the knowledge, skills, and assessment criteria required to earn each badge, ensuring both transparency and credibility. Notably, digital badges also serve as a mechanism for broader engagement: when shared publicly, they act as interactive touchpoints—viewers can click on a badge to learn more or begin their own badge journey, creating a ripple effect that expands awareness and participation in Team Science. Digital badges are particularly well-suited for use within the consortium of a Hub, offering a scalable and standardized approach to address the current fragmentation in Team Science training and professional development. (See Table 1 for a list of the ACTS TSP Digital Badge Value Propositions).

**Table 1:** ACTS TSP digital badge value propositions.

*Flexible and personalized learning*	•Allow professionals to tailor their training to align with specific career goals and areas of interest
•Enables “just-in-time” learning as TSP advances from Fundamentals to Intervention Expert
•Acceptable coursework utilizes innovations developed at translational science Hubs incorporating new discoveries/approaches/training methods
*Modular and stackable credentials*	•Training is broken into smaller, manageable modules that can stand alone or be combined (“stacked”)
	•Learners can build a portfolio of recognized achievements over time
	•Provide adaptability for all CTS roles, from team science to regulatory science
*Verifiable and portable recognition*	•Training is backed by a trusted national organization (ACTS), that standardizes the professional recognition
	•Included metadata from Credly^TM^ makes badges easy to verify, ensuring transparency and credibility for peers, employers, and institutions
	•Badges can be shared across platforms (e.g., LinkedIn, resumes) making them portable and useful in multiple professional contexts.
*Increased visibility and professional development*	•Supports continuous skill development in response to emerging best practices
	•Badges help TSPs stand out to peers, employers, and institutions
	•Standardized credentials help define and reinforce the professional identity of TSPs

## Digital badge initiative

The ACTS TSP Digital Badge Suite currently under development by the TSP SIG aims to establish and evaluate shared standards while strengthening professional identity and institutional legitimacy. The TSP SIG envisions the badges as a way to support changes in the TSP training paradigm at Hubs. Recent studies in the field of education suggest TS Digital Badges could serve as visible professional development markers to colleagues and academic institutions of legitimacy, credibility, and value of Team Science expertise and roles. (Roy and Clark, 2019; Bell et al., 2022; Law et al., 2024) TSP digital badges may also foster a stronger sense of professional identity for TSPs, potentially leading to positive outcomes such as increased retention, reduced burnout, more effective teams, and better Team Science innovations and products (Jones et al., 2018; Monrouxe et al., 2017; Sabanciogullari and Dogan, 2015; Mook et al., 2025).

The digital badge development project began in 2024 with the engagement of Team Science experts from the CTSA Team Science Affinity Group. Members participated in a series of consensus building discussions and literature review critiques to advance a shared understanding of the four initial badges. This was an iterative development of badges based on the expertise and experience of TSPs from 13 Hubs, reflecting on their own careers and professional development. The process was informed by published frameworks, including the 2020 competencies for individuals and teams engaged in translational research (Lotrecchiano et al., 2020). Of particular importance are 1) the intersection of competencies at both the individual and team levels, 2) the utility of the more broadly defined competency domains of facilitation of teams, team communication, collaborative problem solving and team leadership, and 3) the contextual consideration of understanding and working with translational teams, which is the primary intended audience for this suite of digital badges.

With a goal to provide broad relevance to an expected (and confirmed–see **The Fundamentals Badge Pilot**, below) diverse group of professionals and offer practical and meaningful knowledge and skills, a series of four distinct and stackable digital badges were proposed (Table 2).

- The **TS Fundamentals** badge is designed for aspiring TSPs who are new to Team Science. Training opportunities for this badge cover key Team Science principles and introduce individuals to the Science of Team Science (SciTS).
- The TS **Practitioner** badge allows those working in Team Science (e.g., members of translational research teams) to demonstrate their ability to apply SciTS competencies.
- The **Translational Team (TT) Trainer** badge provides training and credentialing for individuals providing Team Science education. Once completed, this badge will verify that such individuals have demonstrated the ability to teach Team Science skills to individual learners, translational teams, or both.
- The **(TT) Intervention Expert** badge verifies that TSPs, educators and/or facilitators can deliver Team Science interventions to enhance translational team performance.

**Table 2:** Team Science Professionals (TSP) digital badge suite.

Certification Level	Competencies	Existing trainings	How to apply	Primary training audience^*^
*Team Science Fundamentals* •Introduce SciTS and key team science principles	Basic SciTS knowledge	° Teamscience.net ° Local TS courses	➢ Take Training ➢ Submit proof of completion	Aspiring Team Science (TS) Professionals, Educators, Scholars, Practitioners, Evaluators, Facilitators
*Team Science Practitioner* •Demonstrate ability to apply SciTS competencies	TT individual-level competencies (Lotrecchiano)	° Team MAPPS ° Local TS courses (as available)	➢ Take Training ➢ Develop Portfolio ➢ Mentor Meeting	TS Practitioners
*Translational Team (TT) Trainer* •Demonstrate ability to teach team science skills to individual learners or TTs	TT individual & team- level competencies	° Team MAPPS ° Local TS courses (as available)	➢ Participate in “Train the Trainer” ➢ Develop Portfolio ➢ Mentor Meeting	TS Educators
*TT Intervention Expert* •Demonstrate ability to deliver TS interventions that enhance team performance	TT individual & team- level competencies	° Collaboration Planning (UW-ICTR) ° Toolbox Dialogue Initiative (MSU) ° Research Jams (MICHR)	➢ Take Intervention Training ➢ Develop Portfolio ➢ Mentor Meeting	TS Practitioners, Educators, and Facilitators

Each badge will leverage educational content developed by Hubs within the consortium [e.g., Teamscience.net for the TS Fundamentals Badge (Spring et al., 2019; Aronoff and Bartkowiak, 2012) or TeamMAPPS (Bisbey et al., 2021; Molldrem et al., 2025) for the TS Practitioner Badge]. The TS Fundamentals badge serves as a prerequisite for all other badges in the suite, providing a shared foundational grounding in team science concepts, language, and knowledge. Badges beyond the “Fundamentals” level offer more robust certification of skills, knowledge, and competencies from TS Practitioner to TT Trainer and TT Intervention Expert. Beyond this common foundational requirement, the badge framework is intentionally flexible, enabling TSPs to tailor their badge portfolio to align with their specific roles, responsibilities, and institutional contexts in supporting translational teams. Table 2 presents, from left to right: illustrative competencies associated with each badge; sample existing training opportunities that support badge attainment; guidance on how to submit evidence of completion; and the intended audience for each badge.

## The fundamentals badge pilot

Initial steps for launching the ACTS TSP Digital Badge Suite involved a pilot for the Fundamentals badge. This pilot initiative was to serve as a mechanism to assess feasibility, usability, and perceived value of the suite of badges using a well-established, competency-focused Team Science training mechanism, the academic modules on teamscience.net (Spring et al., 2019; Aronoff and Bartkowiak, 2012). These free, asynchronous, self-paced modules cover the relevant skills and competencies for the target audience - aspiring TSPs, educators, scholars, practitioners, evaluators, and facilitators. Key skills and competencies covered included:

- Understanding the key characteristics of successful clinical and translational teams
- Differentiating between multidisciplinary, interdisciplinary, and transdisciplinary research
- Appreciating the value of multiple disciplines in addressing complex problems
- Identifying and leveraging unique disciplinary perspectives
- Navigating common challenges and applying appropriate strategies for cross-disciplinary collaboration
- Identifying, utilizing and assessing tools that enable effective team-based research

To receive the Fundamentals badge, users had to successfully complete a minimum of two required modules:

- Science of Team Science – for foundational training in TS principles
- One specialized course – Behavioral Science, Biomedical Science, or Clinical Team Science.

ACTS managed the digital badge submission portal and administrative review of submitted applications. Once a user completed the required modules, they took a screenshot of the two completed modules that included their name as proof of completion. The screenshot was then uploaded into the application submission portal for review. Users were also asked to provide relevant background information and complete a short survey to assess feasibility, usability, and perceived value and impact of the TSP Digital Badge Suite. Successful applicants received an email notification from ACTS containing a link to Credly^TM^, the digital credentialing platform responsible for issuing the badges, where they could complete the final steps to manage and display their digital badge. Any unsuccessful applications received an email notification from ACTS with feedback about how to resubmit successfully. Responses were typically received within 2 weeks of submission.

Thirty-two participants completed the Fundamentals badge during the April to August 2025 pilot. Nine participants were new to Team Science with https://www.teamscience.net/ being their first course. Survey participants included an array of job titles, roles, and levels of Team Science expertise, underscoring the diversity of individuals engaged in translational science and reinforcing the inclusive nature of the TSP community.

One of the goals of the pilot survey was to better understand the feasibility and usability of the digital badge process. Most participants “agreed” or “strongly agreed” that the pilot process was easy, accessible, and clear (4.22 on a 5-point scale from strongly disagree to strongly agree). A second goal was to assess the perceived value of the digital badges. To do this, participants selected potential role-related benefits from a pre-defined list, including greater confidence in Team Science skills, increased credibility as a Team Science practitioner, increased team satisfaction and engagement, and new career pathways. On average, participants expressed interest in pursuing other badges in the suite.

## Next steps

To advance the professionalization and impact of Team Science Professionals (TSPs), several key actions are needed:

- **Institutional Investment in TSP Workforce Development** - Increased support is essential to build sustainable career pathways, standardized credentials, and a recognized professional identity for TSPs. This investment will catalyze the development and dissemination of evidence-based interventions and training programs that are urgently needed across the field of team-based research.
- **Solicitation, review and curation of “eligible” Team Science training and education materials** - A small working group is currently adopting a solicitation and review process. Individuals with existing training materials or programs which may meet eligibility expectations (e.g. learning objectives and competency development) are invited to submit initial information and meet with a team Science Digital Badge consultant (work group member) to collaboratively review materials and assess immediate or upon revision eligibility. We believe this “relationship rich” review process will 1) foster a community of practice within the field of TSPs, 2) increase visibility, awareness and utilization of existing Team Science education and trainings, and 3) identify gaps in resources and opportunities for future development and inter-institutional collaborations.
- **Support for Team Science Scholarship** - Expanding scholarship in Team Science will help generate the theoretical and empirical foundation necessary to guide best practices, inform policy, and elevate the visibility and value of TSPs within the broader research ecosystem.
- **Ongoing Work of the TSP SIG** - The SIG will continue to refine and expand the digital badge system, ensuring that it remains responsive to the diverse roles and career stages of TSPs. Evaluation efforts will focus on understanding how badges influence professional identity, institutional recognition, and individual career progression.
- **Landscape and Impact Assessment** - Insights from the pilot survey informed a broader landscape assessment of Team Science infrastructure across institutions. Therefore, additional measures will strategically assess the impact of digital badges over time. This ongoing evaluation will be critical to understanding the evolving infrastructure available for TSPs, as well as determining if efforts like the digital badge have a meaningful impact on the careers of TSPs.

## Conclusion

The work described herein posits that efforts to professionalize TSPs will enhance capacity across the translational ecosystem, given the pivotal role TSPs play in advancing high-impact, cross-disciplinary research and translation. There is growing recognition that expertise in cross-disciplinary team science is essential to achieve the promised solutions and innovations in complex problem areas; however, limited legitimacy afforded to TSPs continues to restrict recognition of their expertise and constrain their impact (Hoffmann et al., 2022; Hendren and Ku, 2019; Bammer et al., 2020; MacLeod, 2018). To fully realize the potential of this growing workforce, there is an urgent need to build the professional scaffolding that supports TSP success. The Digital Badge Initiative addresses this need by creating standardized, competency-based credentials that validate TSP skillsets and offer structured, yet flexible educational resources to support professional growth. In this way the Digital Badge Initiative will enhance the capacity of the Hubs to effectively support and conduct rigorous translation.

The Digital Badge Initiative serves as a visible, verifiable credentialing system that validates evolving TSP skillsets and reinforces their essential contributions to translational research and science. By raising the visibility of TSP expertise, we aim to empower teams to more effectively integrate and apply team and translational science principles, ultimately amplifying the success and impact of collaborative research.

## Data Availability

The raw data supporting the conclusions of this article will be made available by the authors, without undue reservation.
